# Reemployment premium effect of furlough programs: evaluating Spain’s scheme during the COVID-19 crisis

**DOI:** 10.1186/s12651-023-00343-w

**Published:** 2023-06-07

**Authors:** J. Garcia-Clemente, N. Rubino, E. Congregado

**Affiliations:** 1 Department of Economics, Faculty of Business, University of Huelva, Plaza de la Merced 11, Huelva, 21002 Spain; 2 International University of Andalusia, Seville, Spain; 3 University of Barcelona, Barcelona, Spain

**Keywords:** Furlough, Short-time work, ERTE, Propensity score matching, COVID-19, Spain, J08, J38, J65, J68

## Abstract

This paper presents an average treatment effect analysis of Spain’s furlough program during the onset of the COVID-19 pandemic. Using 2020 labour force quarterly microdata, we construct a counterfactual made of comparable nonfurloughed individuals who lost their jobs and apply propensity score matching based on their pretreatment characteristics. Our findings show that the probability of being re-employed in the next quarter significantly increased for the treated (furlough granted group). These results appear robust across models, after testing a wide range of matching specifications that reveal a reemployment probability premium of near 30 percentage points in the group of workers who had been furloughed for a single quarter. Nevertheless, a different time arrangement affected the magnitude of the effect, suggesting that it may decrease with the furlough duration. Thus, an analogous analysis for a longer (two quarter) scheme estimated a still positive but smaller effect, approximately 12 percentage points. Although this finding might alert against long lasting schemes under persistent recessions, this policy still stands as a useful strategy to face essentially transitory adverse shocks.

## Highlights

- We present a novel contribution that assesses the causal effects of furlough programs using updated microdata from the pandemic period in Spain.
- We found key evidence of a robust and positive average effect (approx. 30 percentage points) of furlough schemes on the probability of being re-employed in the short run.
- However, this positive effect was time-dependent and lessened when the furlough scheme was extended for two consecutive quarters (down to 12 percentage points). This result suggests effectiveness losses with time and advises against long-lasting schemes.

## Introduction

The COVID-19 outbreak caused an unprecedented sanitary crisis worldwide, forcing governments to implement restrictive measures, such as mandatory lockdowns and social distancing. Concerned about a boost in unemployment digits, most countries devised portfolios of coronavirus job retention schemes as a way to temporarily protect employees’ positions while the labour market was adjusting to the shock. Although we can find differences in the eligibility requirements, the degree of coverage, and the duration, these national temporary workforce reduction programs share a considerable number of characteristics.^1^ The purpose of these furlough schemes is to maintain the employer-employee match despite not being working, avoiding a sharp increase of unemployment and the breakup of efficient matches during the temporary shock.

In this paper, we assess the furlough program that was intensively used in Spain during the initial phase of the COVID-19 pandemic in 2020. Spanning our sample of individuals in furloughed and their layoff counterparts and applying propensity score matching to estimate the average effect of furlough schemes on subsequent reemployment probabilities, our findings suggest a significant and positive reemployment premium for the furloughed sample. The magnitude of the effect, however, turns out to be highly dependent on scheme duration.

The debate about the introduction of some sort of furlough programs as an alternative to layoffs has been discussed from long ago (Fitzroy and Hart 1985; Burdett and Wright 1989), being a more equitable solution since they are able to spread the costs of labour adjustment across the workforce, rather than on a small number of workers, as a classical layoff strategy would do (Abraham and Houseman 1994). From a theoretical perspective, the Short-Time Work Schemes (STW) may complement the Unemployment Insurance programs (UI), but as UI, they are not free from introducing distortions in the labour market, mainly via moral hazard issues and hindering reallocation. However, an excess of layoffs during an adverse shock might also be inefficient, and this unwanted effect could be attenuated by using these schemes to maintain valuable labour matches during temporary recessions. (Giupponi et al. 2022) Another theoretical matter of concern is the stabilization power of STWs on aggregate demand. In this regard, Dengler and Gehrke (2021) have found that by reducing the unemployment risk of workers, the precautionary savings motive is mitigated, therefore cushioning the fall of the aggregate demand.

To date, most empirical literature has focused on the effects of STW during the Great Recession, with many authors using this period to conduct research from both the macro- and microlevel. One comprehensive assessment comes from the work of Hijzen and Venn (2011), who made use of data from 19 OECD countries to identify causal effects via a differences-in-differences approach. Their findings suggest program effectiveness on job preservation, especially for Germany and Japan, but heterogeneous effects were found across countries. Additionally, Hijzen and Martin (2013) point out that timing might be crucial, as the positive net effect of furloughs might be nonlinear with respect to subsequent reemployment and job creation. Some time-dependent and nonlinear effects have also been described in Gehrke and Hochmuth (2021). Our article tackles the timing issue by considering two-consecutive quarter furloughs versus the single quarter scheme.

Another strategy is followed by Cahuc and Carcillo (2011) and Boeri and Bruecker (2011), instrumenting STW take-up to control for selection bias and evaluate their potential benefits at the onset of the Great Recession. As a result, both studies agree on the potential benefits of the furlough schemes but warn about the inefficiencies that may appear. We tackle this potential bias selection through the matching of treated and control groups of individuals. Recently, Cahuc et al. (2021) proved that although hampered by the existence of windfall effects, STW is still more cost-efficient at saving jobs than any kind of subsidy. For the same period, Giupponi and Landais (2020) assess the effects of Italian STW schemes in a comprehensive analysis from both firm- and worker-level approaches. They found large and positive effects on headcount employment, arguing in favour of welfare-enhancing effects, especially under temporary shocks, when liquidity constraints and labour market rigidities generate an inefficient excess of layoffs in firms. Essentially, the literature verdict seems to resemble the conclusions drawn by Osuna and García-Pérez (2015) and Osuna and Pérez (2021) from Spain, pointing at the existing trade-off between maximizing job preservation and minimizing deadweight costs and fiscal deficits.

On the other hand, we found few papers that did not find positive effects on labour outcomes from the microlevel. This is the case of Kruppe and Scholz (2014), using German establishment data from the 2008-2010 period. Similarly, Biancardi et al. (2022) show that a more intensive use of STW reduced labour costs and productivity per employee, with no effects on hourly productivity and negative but small effects on firm profits in the short term. Finally, Arranz et al. (2020) use propensity score matching techniques to evaluate the impact of Spain’s furloughs on the subsequent labour status of workers, as we do. Nevertheless, the data and period covered are not the same. In contrast, the authors use longitudinal administrative data for the 2008 recession period and focus on the worker specifically within-firm persistence, unexpectedly finding that treated individuals were less likely to remain working with the same employer years later.

Overall, most of the reviewed literature seems to consider that STW or furloughs may have a positive impact on labour outcomes, but this impact is often conditioned by the nature of the shock, the labour market features and the characteristics of the scheme itself. Furthermore, there is a general concern about the implications of these programs on labour market efficiency. Nevertheless, most recent contributions in the literature from the COVID-19 period appear to have focused on the health related effects of layoffs and other cut off measures, leaving the matter of deciding what consequential effect such policy tools would have on immediate future market outcomes untouched. However, the presumably exogenous and transitory nature of this adverse shock turns the current context into the best case scenario to test the validity of the furlough programs. Our study fills this gap by evaluating Spain’s furlough effect on reemployment probability across 2020. To do so, Spanish Labour Force Survey microdata have been filtered to derive a database of workers who have been matched to calculate the average furlough effect on follow-up labour market outcomes. Within this framework, we aim to test whether being furloughed increases or decreases the likelihood of subsequent employment. As a result, we found a strong and positive re-employability premium for furloughed individuals of nearly 30 percentage points (p.p.) over the counterfactual. Nonetheless, this effect is attenuated to 12.2 p.p. for two quarters extended schemes. To complete the analysis, we included a battery of robustness checks to test the sensitivity of our benchmark results to model selection issues, proving the stability of these results.

The rest of the paper is organized as follows: Sect. 2 introduces the reader to the Spanish institutional framework; Sect. 3 presents a detailed description of the data and sample selection procedure; Sect. 4 focuses on the methodological and technical aspects of the analysis; in Sect. 5, we present our benchmark results for the furlough treatment effects; and finally, our concluding remarks are summarized in Sect. 6.

## Institutional framework

In this paper, we evaluate the impacts of the Spanish furlough program, the so-called temporary employment adjustment schemes (ERTEs). Spain is presented as a suitable case study due to the kind of labour adjustment suffered during the previous Great Recession. In this country, labour adjustment has predominantly been extensive, with collective layoffs being the usual. As a result, no euro country, with the exception of Greece, destroyed more jobs than Spain then, which reached an unemployment rate of 26.94% in the first quarter of 2013.

**Fig. 1:**
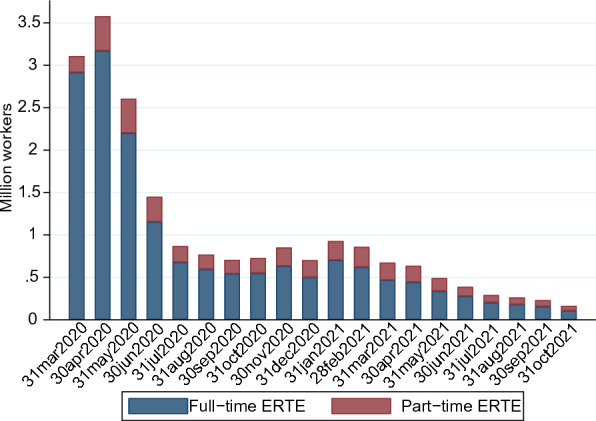
Furloughed workers in Spain during the pandemic. Source: Social Security registers. The bar plot represent how many workers were monthly furloughed from March 2020 to October 2021 in Spain, based on administrative data

Even though the ERTE mechanism already existed by that time (*art. 47, Estatuto de Trabajadores, 1980*), it was only during the pandemic that such a policy tool saw wider application, covering approximately 3 million workers (more than 20% of the affiliated workers) in the second quarter of 2020 (see Fig. 1). In the following quarters, it covered approximately 5% of the affiliated workers, which is still a remarkably higher proportion than it was during the previous recession.

In mid-March 2020, convinced about the transitory nature of the sanitary crisis, the Spanish government quickly entrusted the labour adjustment to fast and wide-coverage COVID-19-related ERTEs (*RD-Ley 8/2020, del 17 de marzo*), encouraging the use of these schemes and imposing penalties on companies that after being granted, were dismissing employees within the next 6 months. This policy, essentially consists of a temporary suspension of the labour relationship between the employer and the employee, or alternatively, a reduction of working hours, justified by a major cause. This cause must be related to economic, technical, organizational or production issues, including COVID-related consequences from March 2020. During this period of suspension, the employee receives a social security allowance while the employer only has to assume a social contribution, which is a minor part of the employee’s wage that sometimes might be even relieved or discharged. As a result, it works as a transitory mechanism of flexibility to adjust the labour market, whose cost is essentially borne by the public administration.

Since its first approval on March 17th, 2020, the expiration date has been postponed several times, remaining in the current legislation and being expected to be redesigned as a permanent employment strategy in the next labour reform. For this reason, some evaluation of the impact of this policy in all dimensions is urgently needed to improve the design of these programs in the future. In addition, we cannot think of a better testing ground for these schemes than the current pandemic scenario, where the shock is strictly exogenous and eminently transitory, and the furlough take-up rate is unprecedented.

In summary, with the aim of tackling this task, this analysis assesses the question of what has been the effect of Spain’s ERTE program on the employees’ follow-up labour outcomes, turning, as far as we know, into the first causal evaluation of these schemes using updated microdata from the pandemic at the individual level. Therefore, our contribution comes from the novelty of the context we have considered, finding evidence of positive effects for furloughed employees on their reemployment prospects in the short term, but strongly conditioned by the duration of the furlough spell.

## Data

Administrative data are the traditional source for conducting this type of analysis; nonetheless, since there is an important delay in its provision, we decided to take advantage of the quarterly flow microdata 2020/q1 to 2020/q4 of the Spanish Labour Force Survey (henceforth SLFS) to perform our analysis. This survey is conducted by the National Statistical Institute and is a large household sample survey providing results on labour participation of people aged 16 and over as well as people outside the labour force in which each sampled individual remains in the survey for a period of six quarters at a time, with no resampling after individuals are rotated out of the sample. The survey is targeted at a rotating sample of approximately 60,000 households throughout the national territory. For every household member, both socioeconomic and labour information is collected to summarize the main characteristics of the Spanish workforce each quarter. As mentioned, individuals in the sample are interviewed for six consecutive quarters; thus, we have information on quarterly labour transitions for a maximum period of 18 months for each individual in the sample.

### Sample selection I: single quarter furloughs

**Fig. 2:**
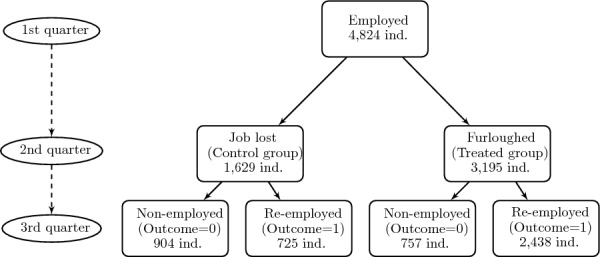
Flowchart for the sample I filtering procedure. The flowchart illustrates the sample selection procedure for the treated and control groups in sample I

As we look for a way to rearrange our data for our initial matching analysis we partly followed the intuition of Izquierdo et al. (2021). First, we filtered our database by selecting those individuals who satisfy these conditions: 1) they consecutively appear in the sample during the first three quarters of 2020, to ensure we can track them in the short term; 2) they were employed during the first quarter of 2020, for the use of comparable pretreatment personal and job characteristics; and 3) they can be identified and divided for treatment during the second quarter, considering those who had lost their job during such period –control group– and those who were full-time furloughed –treated group–.^2^ A binary outcome was finally generated to identify an outcome variable that indicates whether the individual has return to an employment status in the third quarter of 2020, taking value 1 either reincorporated in the former job or a new one. The flowchart displayed in Fig. 2 illustrates the data selection procedure employed for the matching analysis. Therefore, in our final database for this analysis, each observation represents an individual who stayed at least the 3 initial quarters of 2020 in the sample, was employed in the 1st quarter, was either furloughed (treatment group) or displaced/jobless (control group) in the 2nd quarter, and whose employment status was observed in the 3rd quarter (outcome=1 if he or she had been re-employed, outcome=0 otherwise, e.g., still jobless or furloughed). Then, we have an identifier for each individual, a treatment dummy indicating the furloughed in the 2nd quarter, an outcome dummy indicating the reemployment status in the 3rd quarter, and the observable pretreatment characteristics of each individual, thus taking their values in the 1st quarter. Note that the database maintains a cross-sectional structure because each observation represents a single individual with their 1st quarter characteristics, and the time dimension was only used for the treatment assignment and the outcome generation.

This final sample I keeps a total number of 4,824 individuals, with 1,629 in the control group and 3,195 being furloughed for a single quarter. In addition, the proportion of furloughed individuals who were re-employed in the next quarter was 76.31%, compared with only 44.51% in the comparison group, as shown in Table 1.

**Table 1:** Descriptive summary of the outcome (reemployment in the next quarter) by assignment to treatment (furlough program), raw sample I

	Re-employed		Total
	No	Yes	
Unfurloughed	904 (55.49%)	725 (44.51%)	1629 (100.00%)
Furloughed	757 (23.69%)	2438 (76.31%)	3195 (100.00%)
Total	1661 (34.43%)	3163 (65.57%)	4824 (100.00%)

No. of individuals in the sample by outcome and treatment assignment

Percentage over group in parenthesis, aggregated by row

### Sample selection II: two consecutive quarter furloughs

**Fig. 3:**
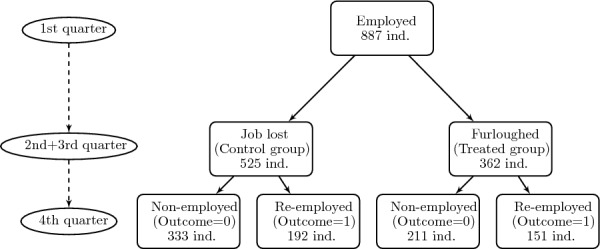
Flowchart for the sample II filtering procedure. The flowchart illustrates the sample selection procedure for the treated and control groups in sample II

We now ask what would happen if we were to aggregate those workers who have been furloughed during both the second and third quarters of 2020 to verify the causal consistency of the impact of the Spanish ERTE on employment in the last quarter of 2020. Hence, the point of this section is to compare the average treatment effect in the previous quarter-to-quarter analysis with an estimate coming from a treatment group that has spent relatively more time being furloughed. We are thus mainly interested in 1) estimating a medium-term effect and 2) establishing a relative comparison in terms of magnitude between the previous and the current exercise. This time, the filtering procedure for the data is analogous. However, now the individuals considered for both the control and treatment groups must necessarily stay in the same situation during the second and third quarters consecutively, as illustrated in Fig. 3. Despite dramatically reducing the sample size, this medium-term analysis still preserves significance.

Table 2 displays the summary of the final 887 individuals who comprise sample II: 525 unfurloughed versus 362 furloughed for the two consecutive quarters. Now, the proportion of re-employed furloughed individuals is not that high, reaching 41.71%, compared to 36.57% for the control group.

**Table 2:** Descriptive summary of the outcome (reemployment in the next quarter) by assignment to treatment (furlough program), raw sample II

	Re-employed		Total
	No	Yes	
Unfurloughed	333 (63.43%)	192 (36.57%)	525 (100.00%)
Furloughed	211 (58.29%)	151 (41.71%)	362 (100.00%)
Total	544 (61.33%)	343 (38.67%)	887 (100.00%)

No. of individuals in the sample by outcome and treatment assignment

Percentage over group in parenthesis, aggregated by row

## Empirical approach

Based on the standard potential outcomes framework for causal evaluation, our approach looks for an average treatment effect by using Propensity Score Matching techniques (PSM), developed in the seminal contribution of Rosenbaum and Rubin (1983). PSM uses as identifying tools a number of observable control variables capable of capturing the relevant differences between groups. As defined in the previous section, we work at the individual level, using a treatment indicator for the furlough and unfurloughed groups, and an outcome dummy that measures the subsequent return to employment. Finally, we match both groups on the individual pretreatment characteristics.

Our first assumption will be based on the concept of conditional independence: once a set of observable variables able to capture all possible forms of heterogeneity has been identified and fixed, this identifying assumption implies that the results of the two groups of units (treated and untreated) have the same potential outcome on average in the population, represented in Expression 1.

1 $$\begin{aligned} E[y_{0}|X,D=1]=E[y_{0}|X,D=0]; E[y_{1}|X,D=1]=E[y_{1}|X,D=0] \end{aligned}$$

where $y_{0}$ is the unsuccessful potential outcome (no return to employment next quarter), $y_{1}$ is the successful potential outcome (return to employment next quarter), *X* is our set of controls and *D* is the assignment to treatment (furlough).

However, the PSM method uses a propensity score (probability of treatment given the *X*) to solve the dimensionality problem when matching on a large set of controls. Thus, the propensity score represents the probability that an individual might be part of the furloughed group given the observables $X_{i}$ and was calculated through a logit regression, as shown in Expression 2.

2 $$\begin{aligned} p(X)=P(D=1|X)=\frac{\exp {(\delta X)}}{1+\exp {(\delta X)}} \end{aligned}$$

The set of observable *X* controls selected for the propensity score calculation can be classified into two categories: social demographics and labour conditions. For the social-demographic dimension, we considered a set of standard controls as sex, age, age squared, region (Spanish Autonomous Community level), education (five levels from primary to higher education) and a foreign dummy. On the other hand, the labour economic dimension comprises the 1st quarter industry or economic activity where the individual was employed (by the 1-digit Spanish National Classification of Economic Activities), combined with 1-digit occupation, type of contract (either temporary or permanent) and the type of working day (either part or full-time).^3^

In addition, any matching procedure essentially requires enough close couples to construct a valid counterfactual in a given neighbourhood of values. An essential way to define the concept of common support has to do with the nonexistence of a full probability set for any given characteristic inside matrix *X*. In other words, the common support requirement implies that for any observable $X_{i}$, the proportion of furloughed individuals with a specific value of that characteristic should always be higher than 0 and less than 1 (Expression 3). The absence of such a condition would imply an empty set for the untreated, and the absence of counterfactual for that specific characteristic $X_{i}$ would immediately bias the estimated values.

3 $$\begin{aligned} 0<P(D=1|X_{i})<1 \end{aligned}$$

Similar values of the propensity score, according to discretionary proximity criteria, are then used to match furloughed workers from the treated with unfurloughed workers from the control group. First, our benchmark matching algorithm will be the straightforward nearest neighbour matching. This algorithm will match any treated individual with his or her nearest counterpart in the control group based on their propensity scores. Furthermore, this starting approximation will not consider any additional constraints, such as calliper options to limit the distance of the couple and no replacement settings. However, all these alternative model specifications are tested and discussed in the appendix, proving the consistency of the benchmark results. More complex algorithms such as kernel matching are also developed and displayed in the appendix section, without any significant change in the magnitude of the result.

Finally, once the samples have been matched, we can estimate the Average Treatment Effect on the Treated (ATT) as the average difference in potential outcomes for the furloughed individuals:

4 $$\begin{aligned} ATT = E[y_{1}|D=1] - E[y_{0}|D=1] \end{aligned}$$

As a methodological digression, we shall mention that, apart from matching, there were alternative methods to control for the selection bias generated in the treatment assignment when it is not random. Typically, the methods that have been widely used with microdata in economic policy evaluations are regression discontinuity design (RDD), differences in differences (DiD) or instrumental variables (IV). Indeed, RDD and DiD are more powerful techniques than PSM since they are able to control for unobserved factors. However, these methods cannot be used for this analysis because the nature of our sample and treatment made it impossible: RDD needs a threshold rule in the continuous range of a certain variable that assigns individuals either to the treated or untreated group (e.g., a grant that is given to the students when their incomes are lower than a threshold value); and DiD needs the outcome variable to be observed before and after treatment (e.g., analysing wages before and after applying a minimum wage policy for similar pretreatment individuals), which is not possible this time because, as we explained before, our outcome variable is generated after treatment by definition, that is, considering whether the individual has been re-employed after being furloughed or not. On the other hand, the IV method makes use of instruments in a first-step regression to estimate the treatment variable, leading to a second regression where the outcome is estimated considering the previous step. Hence, this technique does not substantially differ from PSM since both focus on modelling the treatment through a previous step, using observed controls that may affect the treatment assignment. However, since the scheme was widespread during the pandemic, it is difficult to find a credible exogenous source of variation to use as an instrument for scheme take-up. Overall, we consider that PSM is good enough to infer causality in this particular situation because it perfectly fits the nature of our data and, most importantly, because the treatment assignment should not be affected by unobserved factors since it depends on eligibility criteria, satisfying the main theoretical assumption of the method. To add more to this, it is worth mentioning that PSM has already been used in the literature to control for selection bias when evaluating the same policy in the past (see the example of Arranz et al. (2020)). Additionally, it is worth mentioning that any of these quasiexperimental methods rely on more theoretical principles, such as the stable unit treatment value assumption (SUTVA). Ideally, it requires no spillovers from treated units to untreated and vice versa; thus, the individual outcome should be only affected by its own exposure to treatment and not by others.

## Benchmark results: single versus two-consecutive quarter furloughs

In this section, we offer an overview of our benchmark results, together with pre- and postestimation checks, illustrating how the average treatment effect on the treated stands out as being statistically significant, favouring the furlough scheme as a means of reemployment. Remember we evaluate the transition from a state of furlough to a state of employment on the quarterly basis of 2020, as explained in the data section, leading to an analogous propensity score matching analysis using both samples described. As previously mentioned, our first preliminary look at the data starts with a simple smoothing baseline approach, pairing each treated individual with the nearest neighbour ($k=1$) in terms of propensity score, with no calliper and allowing for replacement. However, all these discretionary decisions were also tested and did not substantially change the benchmark results shown here.^4^

**Fig. 4:**
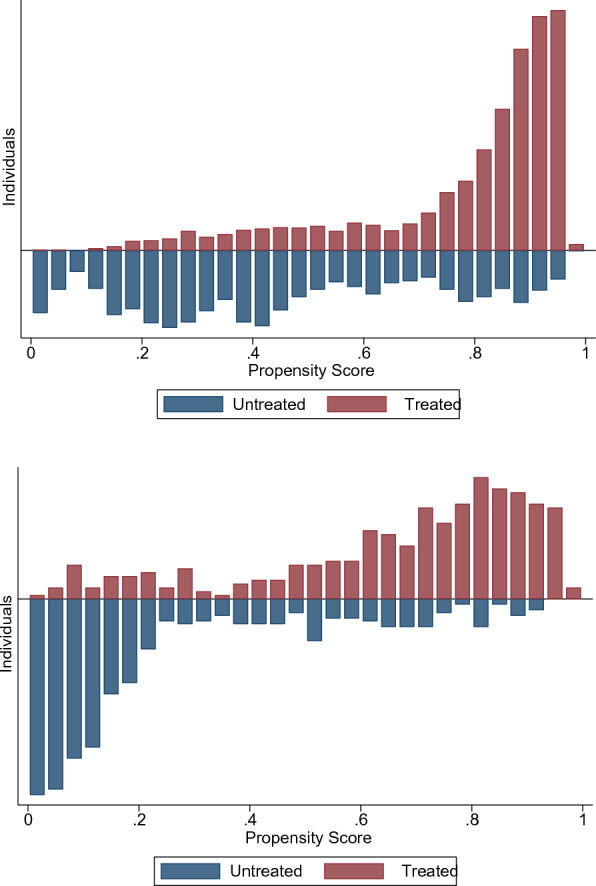
Bar plot of the overall distribution of the propensity score, unmatched samples. The bar plots represent the frequency distribution of the propensity score for treated and untreated groups in both unmatched samples. Ideally, for the common support assumption to be satisfied there must be overlapping individuals along the score distribution. For this reason, the vertical axis is displayed symmetrically

After the logit estimation for the propensity score,^5^ we examine its distribution over the furloughed and nonfurloughed samples (see Fig. 4). This graph evidence the existence of overlapping individuals in the sample, a necessary condition to carry out the subsequent analysis. Then, Table 3 shows our benchmark estimates for the average effect on the treated (ATT) for both samples: single quarter furloughs (ATT_1q) and two-consecutive quarter furloughs (ATT_2q). Both bootstrapped and Abadie-Imbens standard errors^6^ with respective z-stats are displayed together, as it is not fairly clear in the literature which one should be used preferably.

The naive difference between groups leads to an average net effect of 0.317 in the first sample and 0.044 in the second. However, after matching we ended up with an average treatment effect on the treated (ATT) of 0.294 for single quarter furloughs and 0.122 for two quarters, which can be interpreted as a premium of 29.4 percentage points (hereafter p.p.) in the probability of being re-employed thanks to the single quarter furlough scheme, and 12.2 p.p. when the scheme lasts for two quarters. These coefficients have been tested significantly different from zero based on both methods considered for estimating the standard errors.

These results suggest that the effect on reemployment for two quarter schemes is smaller in magnitude when compared to one quarter duration, which may favour the idea of effectiveness losses in the furloughs schemes when they are extended. Remember that a single quarter scheme had an ATT that was approximately 17 p.p. above the estimated effects for the two-consecutive quarter. Be that as it may, the positive causal effect of furloughs on employment appears to have helped workers in Spain across the whole year, regardless of the duration of the furlough policy.

**Fig. 5:**
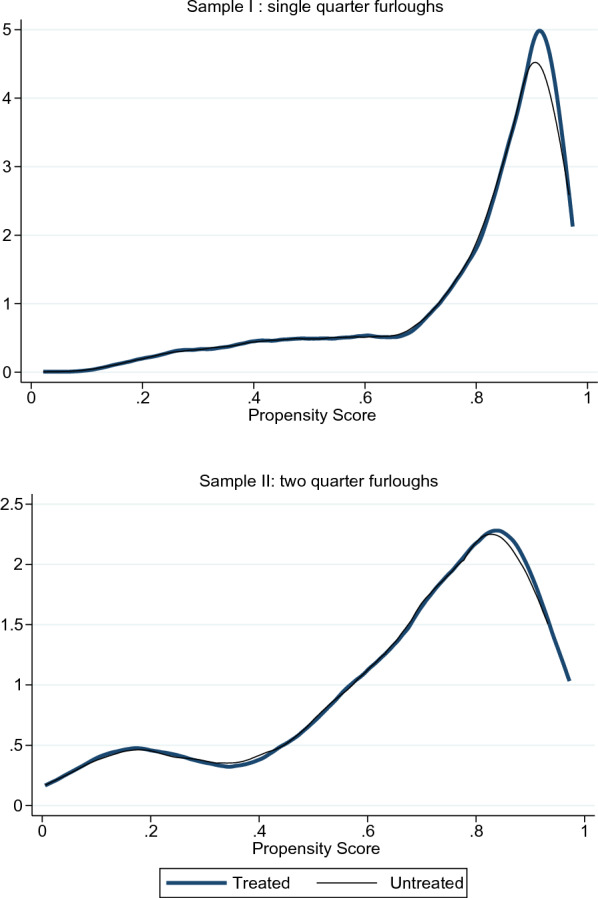
Densities of the propensity score after matching. It represents the propensity score density distribution after matching. Ideally, a successful matching achieves similar densities for treated and untreated groups

**Fig. 6:**
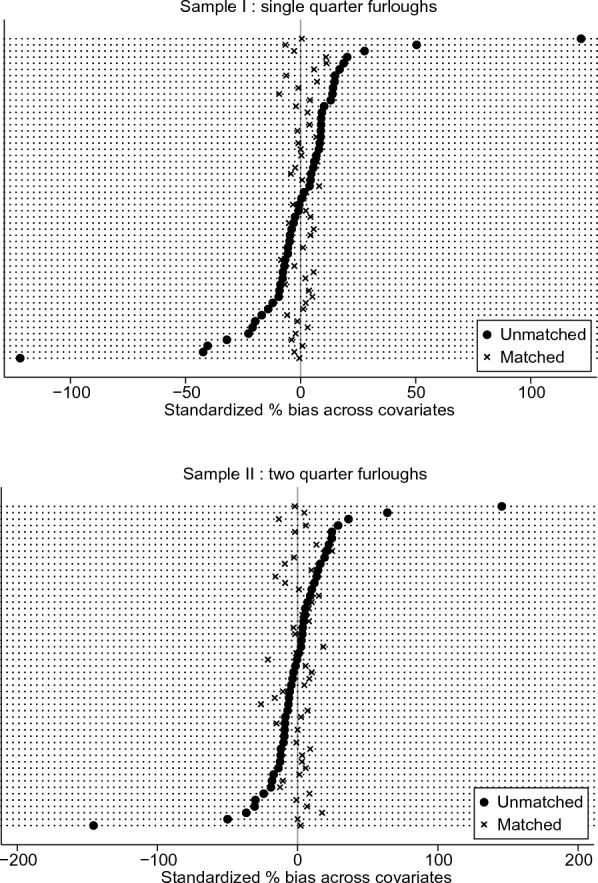
Standardized percentage bias across covariates before-after matching. Relative bias between treated and untreated for any matching variable used for the propensity score. Ideally, we would expect a bias reduction for most of the covariates after matching

To infer the (joint) validity of the matching procedure, Fig. 5 shows the score densities before and after matching. Most strikingly, the two distributions appear to be almost identical after the procedure. This last result is further enhanced by the bias reduction plot in Fig. 6: after matching, the average bias is clearly reduced and less dispersed around zero.^7^ Judging by these results, the matching procedure succeeded in balancing the treated and untreated, leading to a very similar distribution in terms of propensity score. Additional summary statistics for this procedure are available in Table 4, showing a clear reduction of the matching variables in their ability to explain the treatment assignment after matching (measured by the pseudo R-squared), together with a noticeable decrease in the mean and median bias for the matched samples, as is desirable.

**Table 3:** Propensity score matching benchmark results: average treatment effects on the treated for single quarter furloughs (1q) and two-consecutive quarters furloughs (2q)

*Estimand*	*Coefficient*	*BS S.E. (z stat)*	*AI S.E. (z stat)*
(I)	(II)	(III)	(IV)
*Unmatched_1q*	0.317		
*ATT_1q*	0.294	0.0287 (10.24)	0.0304 (9.66)
*Unmatched_2q*	0.044		
*ATT_2q*	0.122	0.0572 (2.13)	0.0410 (2.98)

(I) Estimands, (II) estimated values, (III) 500 bootstrapped standard error and (IV) Abadie-Imbens standard error, with associated z stats in parenthesis

**Table 4:** Propensity score matching quality checks

*Sample*	*Pseudo R* $^{2}$	*LR * $\chi ^{2}$	*p* $> \chi ^{2}$	*Mean bias*	*Median bias*
(I)	(II)	(III)	(IV)	(V)	(VI)
*Unmatched_1q*	0.282	1642.98	0.000	16.3	8.9
*Matched_1q*	0.016	141.75	0.000	4.0	3.6
*Unmatched_2q*	0.365	422.55	0.000	19.7	11.9
*Matched_2q*	0.078	77.02	0.001	8.4	8.0

Values of the pseudo R-squared coefficient and related tests on the joint hypothesis of non-significance of the control variables in (II), (III), and (IV). Mean and median bias in column (V) and (VI). Ideally, we would expect significant reductions in the bias measures and the pseudo R-squared after matching

## Conclusions

We used the 2020 quarterly waves of the Spanish Labour Force Survey to collect a sample of workers who were furloughed during the initial phase of the pandemic, together with comparable nonfurloughed individuals who lost their job at that time and joined any source of potential workforce, using the latter to construct the counterfactual. Then, performing propensity score matching techniques, we provide evidence on how the probability of being re-employed was significantly higher in the treated group (furlough granted group) than in the control group, indicating a positive net effect on after-furlough re-employability near 30 percentage points. This result seems consistent with previous findings of Giupponi and Landais (2020) for the Italian STW effect on workers’ reemployment probability when using a layoff counterfactual as we did. Additionally, our analysis has found results that are robust to a variety of alternative specifications, which may be a timewise different data arrangement or a series of tweaks to the selection procedure related to the matching method. Nonetheless, the magnitude of the treatment effect decreased significantly when two-consecutive quarter schemes were considered in comparison to the single quarter scheme, which supports the idea of furlough effectiveness losses when they are extended. Considering the reviewed literature, such similar results for analogous schemes in other countries during the previous recession suggest potential external validity for our findings. Most likely, when the scheme is transitory, it is able to maintain the efficient labour matches that otherwise would not endure, prevailing this positive effect. Conversely, a long-lasting scheme may uncover the inefficient aspects of this policy: it might target less efficient matches that are mainly affected by structural labour market changes and therefore hinder the necessary reallocation of workers. As a result, although these job retention programs seem to be a useful strategy to face transitory adverse shocks, one might expect that when a shock is of a more permanent nature, these schemes only delay the destruction of jobs. Extending the analysis to more enduring schemes to test whether their effects keep shrinking and vanish with time would be interesting, but data nonavailability prevents us from doing so. Likewise, analysing the long-term effects of the programs is an unresolved matter for further research.

To conclude, intuition tells us that when many jobs were suspended due to lockdown and social distancing measures at the beginning of the pandemic, these short-time work schemes did a great job of preserving the workers’ position while favouring labour market adjustment at the lowest cost for economic agents. Conversely, jobs affected by more structural changes will probably be captured in a furlough program for a long time, wasting public resources and generating deadweight losses while hindering the workforce reallocation process. Therefore, as an implication for policy-making, STW public schemes appear to be once more a very relevant policy tool when labour market stability is the target as long as the shock is expected to be transitory. However, any public choice related to this kind of tool should be considered, keeping in mind that the duration and timing of the manoeuvre is essential for it to reduce social costs and achieve the highest possible effect on re-employability, given the conditions of the labour market in the COVID era. Future research may continue exploring this topic with new data, trying to overcome some of the limitations that we have faced, looking for long-term and dynamic effects, using other identification strategies and identifying some of the heterogeneity sources.

## Figures and Tables

**Fig. 7:**
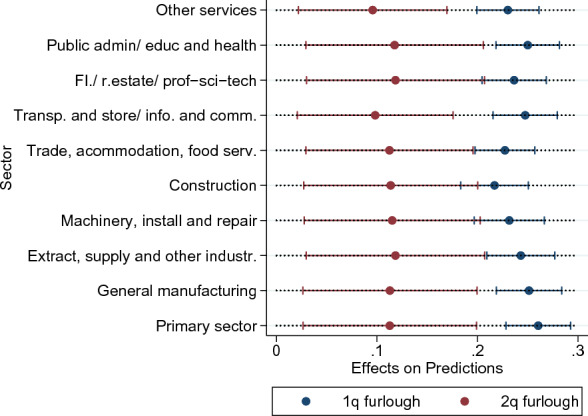
Computed average marginal effect of furlough on the reemployment probability with a 95% confidence interval by sector

**Fig. 8:**
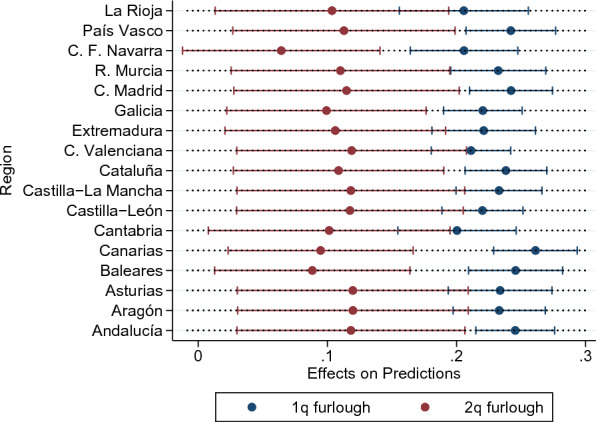
Computed average marginal effect of furlough on the reemployment probability with a 95% confidence interval by region

## Data Availability

The datasets generated and analysed during the current study will be available in the corresponding author’s website https://javier-garcia-clemente.weebly.com/materials.html, or alternatively, upon reasonable request.

## Appendix A: Robustness checks and heterogeneous effects

### Calliper, more neighbours, no replacement and kernel matching

To prove the robustness of the results previously seen, this section presents and compares an alternative matching choice to the benchmark we already established with the k=1, no calliper approach with replacement. First, we will produce results based on different discretionary choices of the calliper. We note that the reason why a calliper can be imposed on this kind of discretionary procedure is that some treated individuals could be very far away from the closest untreated individual. That would imply a reduction in the matching precision as treated individuals might be paired with dissimilar untreated ones. Having a calliper is a way to ensure the existence of a common support interval, but the lower its value is, the higher the chances of leaving some individuals out of the estimates. Thus, while in the benchmark case the loss of comparable individuals was 0, when imposing a calliper we may lose some individuals who might be off support, which means, out of the maximum score distance we have established with the calliper. In exchange, we will ensure that any paired individuals are as similar as we desire in terms of their propensity score. Equivalently, a trade-off exists when matching techniques with replacement are compared, everything else equal, to their no-replacement counterpart. Replacement ensures lower bias and higher matching quality because the distance between propensity scores is minimal since the best optimal choices from the control group are not systematically ruled out of the matching procedure. On the other hand, avoiding replacement reduces variances, as all the available information is being employed, but naturally worsens the quality of the matching, as less likely individuals are paired by their less similar propensity scores. One last discretionary choice comes from the number of nearest comparable neighbours used for the matching procedure (*k*). Thus far, our baseline approach has taken only the nearest individual ($k=1$), but the selection of a higher number is equally possible. This option also trades a variance reduction, from the use of more information to construct the counterfactual for each subject, with a bias increase, due to average poorer matches.

As these decisions might cast a shadow on the (internal) validity for the results, this section presents some alternative robustness exercises where a 1% calliper and a more restrictive calliper of 0.1% is used, alongside the usual k=1 and k=10 choice, and replacement and no replacement alternatives to test the robustness of the ATT results under these conditions. Table 5 offers the estimates for: (i) 1 nearest neighbour, 1% calliper, replacement model; (ii) 1 nearest neighbour, 0.1% calliper, replacement model; (iii) 10 nearest neighbours, 1% calliper, replacement model; and (iv) 1 nearest neighbour, 1% calliper, no replacement model.

Additionally, we considered the possibility of allowing for heterogeneous weights in the matching procedure. That is, instead of averaging out the k nearest neighbours, we want to resort to a method that creates a comparable result as a weighted average based on some function. Kernel densities can thus be used so that the comparable result (the propensity score associated with the $\hbox {i}^{th}$ treated) is the weighted average of the propensities of the untreated neighbours with weights negatively proportional to the distance between the propensity score of the $\hbox {i}^{th}$ treated and the $\hbox {j}^{th}$ matched untreated individual. The further away they are, the lower is the contribution to the propensity score computation. An application of the kernel algorithm can be seen in Heckman et al. (1997). We tested two different kernel functions (normal and Epanechnikov), and two different bandwidth values (0.6 and 0.1).

Table 5 shows the on support remaining individuals for each model discussed, together with the ATT results for both analyses (single- and two-quarter furloughs). The 1% calliper imposition only left out few individuals compared to the benchmark case, which evidence the existence of overlapped individuals in both groups, satisfying the common support assumption. Going a step further, we imposed a stricter 0.1% calliper. This time, although the sample loss was higher, the ATT results remain close to the benchmark for the single-quarter analysis, and preserve the sign for the two-quarter analysis. Finally, the no replacement option, together with 1% calliper, leaves out of the pairing procedure many furloughed workers from upper regions of the propensity score, too many when compared to their comparable equivalent in the unfurloughed group. This phenomenon can be explained by the larger number of treated individuals in the sample. In spite of the significant loss, the left sample seems to be enough to reduce the variance of the estimate, even if slightly, while preserving the magnitude of the effect. Last, the kernel results stayed absolutely in line with the baseline case and the imposition of the common support condition did not dramatically affect the remaining sample.

Overall, estimates of the average effect remained close to the benchmark case in every single attempted estimation; therefore, the idea that furloughs might have a positive impact on reemployment opportunities still stands.

**Table 5:** Results for alternative matching models

*Model*	*Group*	Single quarter		Two quarters	
		*On support*	*ATT (BS SE)*	*On support*	*ATT (BS SE)*
(I)	(II)	(III)	(IV)	(V)	(VI)
*k=1, cal=0.01, repl.*	*Untreated*	1447		444	
	*Treated*	3102	0.294 (0.0287)	333	0.114 (0.0535)
*k=1, cal=0.001, repl.*	*Untreated*	1219		153	
	*Treated*	2920	0.290 (0.0275)	144	0.069 (0.0800)
*k=10, cal=0.01, repl.*	*Untreated*	1447		444	
	*Treated*	3102	0.304 (0.0279)	333	0.139 (0.0493)
*k=1, cal=0.01, norepl.*	*Untreated*	964		159	
	*Treated*	936	0.259 (0.0210)	155	0.110 (0.0532)
*epanech., bw=0.06, cs.*	*Untreated*	1493		479	
	*Treated*	3088	0.301 (0.0253)	333	0.139 (0.0515)
*epanech., bw=0.01, cs.*	*Untreated*	1436		434	
	*Treated*	3088	0.305 (0.0262)	321	0.116 (0.0496)
*normal, bw=0.06, cs.*	*Untreated*	1493		479	
	*Treated*	3088	0.298 (0.0240)	333	0.137 (0.0513)
*normal, bw=0.01, cs.*	*Untreated*	1493		479	
	*Treated*	3088	0.307 (0.0257)	333	0.141 (0.0494)

(I) Model specs –*k* no. of neighbours, calliper, replacement, kernel type, *bw* bandwidth and common support–, (II) group, (III) and (V) remaining subsample on support, (IV) and (VI) Average Treatment Effect on the Treated with 500 bootstrapped standard errors in parenthesis

### Heterogeneous effects: regional and sectoral differences

Our main results proved how the analysis of the furlough reemployment effect at the national aggregated level led us to conclude that furloughed individuals on average showed a higher probability of reemployment with respect to unfurloughed workers during the most relentless, initial phase of the coronavirus outbreak. We now evaluate whether any heterogeneity is left at a more disaggregated territorial and sectoral level. However, since the common support assumption cannot be held at these lower strata and other problems using the matching procedure emerge, we will rely on the estimation of a simple logistic regression for the heterogeneity analysis. This exercise was carried out for both sample arrangements that were used before, again with the reemployment binary outcome, and including the treatment dummy with the rest of the covariates considered thus far as independent variables in a regression framework, thus estimating the following regression:

5 $$\begin{aligned} P(Reemployed=1|X,D)=G(\alpha + \beta X + \delta D) \end{aligned}$$

where *G*(.) is the logistic functional form, *X* is the set of personal and labour controls used in the matching analysis, and *D* is the treatment (furlough) indicator.

As the logit coefficients cannot be straightforwardly interpreted and we are particularly interested in the marginal effects of the furlough variable, we will directly focus on its computed average marginal effect in each region and sector to uncover some of the heterogeneity under the average treatment effect. The estimated marginal effects of the furlough program on the reemployment probability by sector and region are displayed in Figs. 7 and 8.

These results overall point at slight although not statistically significant heterogeneity in the treatment effect by sector and region when the same furlough scheme is considered, with all the coefficients close in magnitude to the aggregated value. Nonetheless, they certainly revealed a significant reduction of the marginal effect between the single quarter versus the two quarter scheme effect in most regions and sectors, reinforcing the previous result.

## Appendix B: Supplementary tables

See Tables 6 and 7

**Table 6:** Descriptive summary of the matching variables, raw samples

	Sample I means				Sample II means			
	Treated	Control	%bias	t test	Treated	Control	%bias	t test
(I)	(II)	(III)	(IV)	(V)	(VI)	(VII)	(VIII)	(IX)
Male	48.70	51.14	− 4.9	− 1.60	43.37	50.00	$-$13.3	$-$1.94*
Female	51.30	48.86	4.9	1.60	56.63	50.00	13.3	1.94*
Andalucía	12.02	23.76	− 31.0	− 10.64***	15.75	28.16	$-$30.3	$-$4.35***
Aragón	4.32	3.13	6.3	2.01**	3.32	2.68	3.7	0.55
Asturias	2.66	1.96	4.6	1.49	2.21	1.92	2.1	0.30
Baleares	2.94	1.78	7.7	2.42**	4.70	1.34	19.7	3.03***
Canarias	6.26	3.62	12.2	3.85***	12.98	3.26	36.1	5.58***
Cantabria	2.13	2.03	0.7	0.24	0.83	1.34	$-$4.9	$-$0.71
Castilla-León	10.08	8.04	7.1	2.29**	7.74	8.05	$-$1.2	$-$0.17
Castilla-LaMancha	4.66	6.08	$-$6.3	$-$2.10**	5.80	5.75	0.2	0.03
Cataluña	12.74	8.17	15.0	4.78***	15.47	9.00	19.8	2.96***
C.Valenciana	8.67	9.82	$-$4.0	$-$1.32	5.80	9.00	$-$12.2	$-$1.76*
Extremadura	1.66	3.62	$-$12.3	$-$4.29***	1.93	3.26	$-$8.3	$-$1.19
Galicia	13.80	10.99	8.5	2.76***	9.12	9.96	$-$2.9	$-$0.42
C.Madrid	5.76	6.63	$-$3.6	$-$1.20	6.08	7.47	$-$5.5	$-$0.80
R.Murcia	2.72	3.44	$-$4.1	$-$1.38	3.04	2.87	1.0	0.14
C.F.Navarra	3.22	2.09	7.1	2.25**	1.11	1.53	$-$3.7	$-$0.54
PaísVasco	4.57	2.89	8.9	2.82***	3.32	2.68	3.7	0.55
LaRioja	1.50	1.54	$-$0.3	$-$0.09	0.83	1.72	$-$8.0	$-$1.13
Ceuta	0.16	0.18	$-$0.7	$-$0.22				
Melilla	0.13	0.25	$-$2.8	$-$0.97				
Primary	4.41	9.39	$-$19.7	$-$6.87***	4.42	10.54	$-$23.4	$-$3.31***
Lower secondary	32.65	36.40	$-$7.9	$-$2.61***	30.39	38.12	$-$16.3	$-$2.38**
Upper secondary	16.43	13.75	7.5	2.43**	18.51	13.99	12.3	1.81*
Post secondary	13.37	11.42	5.9	1.92*	13.54	10.73	8.6	1.27
Higher educ	33.15	29.04	8.9	2.90***	33.15	26.63	14.3	2.10**
Age	39.94	37.65	19.2	6.35***	41.03	38.40	22.3	3.22***
Age squared	1731.2	1566.2	17.7	5.83***	1804.0	1629.7	18.8	2.74***
National	91.96	86.25	18.4	6.28***	91.44	88.51	9.8	1.41
Foreign	8.04	13.75	$-$18.4	$-$6.28***	8.56	11.49	$-$9.8	$-$1.41
Indefinite	79.14	27.20	121.9	39.47***	84.72	26.18	145.6	20.66***
Temporary	20.86	72.80	$-$121.9	$-$39.47***	15.28	73.82	$-$145.6	$-$20.66***
Full-time	75.21	71.15	9.2	3.04***	75.41	71.46	9.0	1.30
Part-time	24.79	28.85	$-$9.2	$-$3.04***	24.59	28.54	$-$9.0	$-$1.30
Primary sector	0.31	8.10	$-$39.5	$-$15.51***	0.28	11.11	$-$48.0	$-$6.49***
General manufacturing	3.60	5.03	$-$7.1	$-$2.38**	3.04	4.98	$-$9.9	$-$1.42
Extract, supply, otherIn	4.54	4.24	1.5	0.48	2.49	4.79	$-$12.3	$-$1.75*
Machinery, install, repa	7.29	3.81	15.3	4.79***	4.14	3.45	3.6	0.54
Construction	4.16	10.19	$-$23.5	$-$8.27***	3.04	7.09	$-$18.5	$-$2.62**
Trade, Accomm, FoodServ	49.64	26.21	49.7	16.01***	55.53	26.25	62.3	9.21***
Transp, Store, Info &Comm	5.98	7.55	$-$6.3	$-$2.09**	6.91	7.09	$-$0.7	$-$0.10
FI,RealEstate, Prof-sci	6.89	8.90	$-$7.5	$-$2.50**	7.18	10.15	$-$10.6	$-$1.52
PublicAdmin, Educ, Healt	8.14	14.00	$-$18.8	$-$6.41***	6.63	15.33	$-$28.1	$-$3.98***
Other services	9.45	11.97	$-$8.1	$-$2.72***	10.77	9.77	3.3	0.48
Managerial	1.91	0.86	9.0	2.79***	1.38	0.77	6.0	0.89
Technician: Sci &Intelec	5.26	7.31	$-$8.4	$-$2.84***	5.25	7.47	$-$9.1	$-$1.31
Technician: Support	11.55	7.74	12.9	4.14***	10.50	7.28	11.3	1.68*
Clerical	12.05	7.92	13.8	4.41***	17.68	7.47	31.1	4.70***
Face to face services	35.93	23.45	27.6	8.88***	33.70	23.18	23.5	3.47***
HighSkilled:PrimarySec	0.16	1.41	$-$14.3	$-$5.44***	0.28	1.34	$-$11.9	$-$1.64
HighSkilled: industry	10.96	14.49	$-$10.6	$-$3.56***	6.35	12.45	$-$21.0	$-$2.99***
Factory worker	9.77	8.59	4.1	1.32	7.18	8.24	$-$4.0	$-$0.57
Elementary occup	12.43	28.24	$-$40.1	$-$13.85***	17.68	31.80	$-$33.1	$-$4.76***

* $$p<0.1$$, ** $$p<0.05$$, *** $$p<0.01$$

(I-II, VI-VII) Percentage distribution of the categorical variables between treated and control groups (mean if

continuous –age and age squared–), (III, VIII) percentage bias and (V, IX) t test for unbalanced samples.

Note: Regions Ceuta and Melilla have no observations in sample II

**Table 7:** Logit regression output for the propensity score calculation (probability of treatment)

Variable	Sample I		Sample II	
	Coeff	Z-stat	Coeff	Z-stat
Male	0.109	(1.22)	0.0855	(0.38)
Female	Omitted	(.)	Omitted	(.)
Andalucía	$-$0.433	($-$0.51)	0.559	(0.64)
Aragón	0.320	(0.37)	0.864	(0.88)
Asturias	0.841	(0.94)	1.330	(1.24)
Baleares	0.641	(0.72)	1.833	(1.73)
Canarias	0.603	(0.69)	2.177	(2.38)***
Cantabria	0.00348	(0.00)	0.228	(0.18)
Castilla-León	0.242	(0.28)	0.877	(0.98)
Castilla-LaMancha	$-$0.0920	($-$0.11)	1.030	(1.13)
Cataluña	0.453	(0.53)	1.255	(1.43)
C.Valenciana	$-$0.0984	($-$0.11)	0.563	(0.63)
Extremadura	$-$0.621	($-$0.70)	0.957	(0.91)
Galicia	0.275	(0.32)	0.656	(0.74)
C.Madrid	$-$0.261	($-$0.30)	0.0617	(0.07)
R.Murcia	$-$0.207	($-$0.23)	1.837	(1.81)
C.F.Navarra	0.127	(0.14)	0.488	(0.42)
PaísVasco	0.577	(0.66)	1.390	(1.38)
LaRioja	0.0165	(0.02)	Omitted	(.)
Ceuta	0.743	(0.64)		
Melilla	Omitted	(.)		
Primary	$-$0.297	($-$1.58)	$-$0.823	($-$1.68)
Lower secondary	$-$0.0594	($-$0.53)	$-$0.493	($-$1.74)
Upper secondary	0.120	(0.94)	$-$0.166	($-$0.55)
Post secondary	0.0726	(0.54)	$-$0.123	($-$0.36)
Higher educ	Omitted	(.)	Omitted	(.)
Age	$-$0.00928	($-$0.43)	0.0846	(1.55)
Age squared	0.000129	(0.46)	$-$0.000954	($-$1.37)
National	0.451	(3.43)***	0.550	(1.68)
Foreign	Omitted	(.)	Omitted	(.)
Indefinite	2.205	(26.18)***	2.545	(11.94)***
Temporary	Omitted	(.)	Omitted	(.)
Full-time	$-$0.123	($-$1.31)	$-$0.149	($-$0.65)
Part-time	Omitted	(.)	Omitted	(.)
Primary sector	$-$1.606	($-$3.92)***	$-$2.334	($-$2.08)***
General manufacturing	$-$0.381	($-$1.71)	$-$0.676	($-$1.19)
Extract, Supply, OtherIndustry	0.0260	(0.12)	$-$1.068	($-$1.85)
Machinery, Install, Repair	0.716	(3.28)***	0.245	(0.41)
Construction	$-$0.421	($-$2.03)***	$-$0.602	($-$1.07)
Trade, Accommodation, FoodServ	0.798	(5.92)***	0.516	(1.59)
Transp, Store, Info &Comm	$-$0.151	($-$0.79)	$-$0.467	($-$0.97)
FI, RealEstate, Prof-sci-tech	$-$0.0148	($-$0.08)	$-$0.746	($-$1.77)
PublicAdmin, Educ, Health	$-$0.206	($-$1.26)	$-$0.985	($-$2.31)***
Other services	Omitted	(.)	Omitted	(.)
Managerial	0.698	(1.70)	0.290	(0.34)
Technician: sci &Intelectual	0.720	(3.52)***	0.364	(0.71)
Technician: support	0.951	(5.64)***	0.317	(0.77)
Clerical	0.783	(4.94)***	0.621	(1.67)
Face to face services	0.706	(5.77)***	0.201	(0.71)
High skilled: Primary sector	$-$1.038	($-$1.57)	$-$1.034	($-$0.62)
High skilled: industry	0.555	(3.35)***	0.0854	(0.20)
Factory worker	0.624	(3.63)***	$-$0.0744	($-$0.17)
Elementary occup	Omitted	(.)	Omitted	(.)
Constant	$-$1.556	($-$1.61)	$-$4.578	($-$3.18)***
Observations	4613		849	
Pseudo $R^{2}$	0.281		0.366	
chi2	1642.1***		423.7***	

*z* statistics in parentheses

* $$p<0.1$$, ** $$p<0.05$$, *** $$p<0.01$$

Note: Regions Ceuta and Melilla have no observations in sample II

## Footnotes

1 In this context, furlough schemes and short-time work are the most common terms to refer to these job retention programs, but we will use any term equally throughout this paper. Similar programs in other countries are called *Furlough schemes* in the UK, *Kurzabeit* in Germany, *Activité partielle* in France, and *Cassa Integrazione Guadagni* in Italy. A detailed description of these schemes and their use across Europe can be found in Drahokoupil and Müller (2021).

2 Note that in the control group, we are considering individuals who stopped working in the 2nd quarter, regardless of whether they were unemployed, discouraged or potential workforce that quarter. Since lockdown and containment measures may have significantly hampered the employment active seeking process, we made no distinction between these labour states, as suggested by the Spanish Central Bank’s aforecited report (Izquierdo et al. 2021).

3 Including some other additional labour information on earnings and firm characteristics would enhance the performance of the matching estimator; unfortunately, further details on economic activity (lower disaggregation), earnings or firm identifiers were not available in the dataset.

4 Alternative model specifications and heterogeneous effects are tested in Appendix A. There, we show and discuss the sensitivity of our results to the number of selected neighbours, calliper options, replacement, alternative matching algorithms and regional and sectoral heterogeneity. All in all, the same results are drawn.

5 Logit output available in Appendix B.

6 According to the methods derived by Abadie and Imbens (2006, 2011, 2012)

7 An average bias reduction does not necessarily imply that every single control led to a bias reduction. As a further indication of this, the likelihood ratio test in Table 4 for the matched analysis did not reject the joint null any better than in the unmatched analysis. This, however, a calculated risk entailed having to resort to a high number of covariates to check for any sort of observable heterogeneity, exposing the analysis to the risk of overcontrolling it.
